# A Model for Selecting Kiwifruit (*Actinidia eriantha*) Germplasm Resources with Excellent Fruit Quality

**DOI:** 10.3390/foods13244014

**Published:** 2024-12-12

**Authors:** Lu Chen, Yansong Liu, Huan Gao, Jiale Cao, Jiquan Qian, Kexin Zheng, Dongfeng Jia, Zhu Gao, Xiaobiao Xu

**Affiliations:** 1 Jiangxi Key Laboratory of Subtropical Forest Resources Cultivation, College of Forestry/Landscape Architecture and Art, Jiangxi Agricultural University, Nanchang 330045, China; chenlu@jxas.ac.cn; 2 College of Agronomy, Kiwifruit Institute, Jiangxi Agricultural University, Nanchang 330045, China; 3 Institute of Biological Resources, Jiangxi Academy of Sciences, Nanchang 330096, China

**Keywords:** *Actinidia eriantha*, fruit quality, germplasm resources, comprehensive evaluation, key traits

## Abstract

The evaluation of quality traits is an important procedure for kiwifruit breeding and comprehensive utilization. The present study aimed to establish a comprehensive system to assess *Actinidia eriantha* germplasms by analyzing 22 quality traits on kiwifruit samples collected from a wild population of 236 plants grown in the Jiangxi Province, China. Variability, correlation, principal components, and cluster analyses were carried out using the data collected from fruit quality evaluations. The coefficients of variation (CV) of fruit quality traits ranged from 11.66 to 66.16% (average coefficient = 35.09%), indicating a high level of variation among the 236 plants. In addition, different degrees of correlations were found between the traits, with similar traits demonstrating strong correlations. Principal component analysis (PCA) generated eight comprehensive and independent principal components, accounting for 77.93% of the original fruit quality information. Furthermore, an extensive evaluation from PCA ranked the plants based on cluster analysis and grouped them into seven categories. A stepwise regression analysis generated a prediction model, demonstrating a good fit (0.945) with the principal components of the comprehensive evaluation score. Overall, this study identifies nine quality traits, representing fruit appearance, sweetness, acidity, flavor, and nutritional attributes, as important traits for a comprehensive evaluation of *A. eriantha* fruits.

## 1. Introduction

Kiwifruit (*Actinidia* spp.), also known as the ‘king of fruit’, is one of the world’s most consumed fruits. It has a unique sweet and sour flavor and is rich in a variety of vitamins and minerals [1]. Under the pressure of long-term evolution and natural selection, *Actinidia* spp. has produced abundant phenotypic and genetic diversity within and between species to adapt and survive. China, with its vast reserves and genetically diverse wild resources of *Actinidia* spp., has contributed largely to kiwifruit production. However, the rapid growth of the global kiwifruit industry has significantly increased the demand for diversified varieties with multi-resistance and excellent fruit quality [2]. Typically, the rich genetic diversity provides a robust foundation for developing novel and improved varieties [3]. Breeding for excellent varieties through wild resources selection is one of the main approaches used to develop novel varieties.

China has rich and extensive kiwifruit germplasm resources, and is thus considered a natural kiwifruit genebank [4]. Out of the 75 taxa (54 species, 21 varieties) of the genus *Actinidia*, 73 are found in China. The Jiangxi Province is one of the areas in China with abundant wild *Actinidia* germplasm resources. Many excellent *A. chinensis* cultivars, including ‘Jintao’ [5], ‘Wuzhi 3’ [6], ‘Jinxia’ [7], ‘Jinzao’ [8], ‘Lushanxiang’ [9], and ‘Moshan 4’ [10], and *A. eriantha* cultivars, including ‘Sweet White’ [11] and ‘Ganlv 1’ [12], have been developed from these wild *Actinidia* resources. However, the protection of the resources and the development of excellent germplasm need to be further improved. In long-term evolution, kiwifruit has formed rich phenotypic and genetic diversity among or within species, extensively promoting scientific research, industry development, and progress. With the rapid development of the worldwide kiwifruit industry, the demand for germplasm resources with diversified varieties, excellent quality, and multi-resistance has increased significantly [13]. The rich genetic diversity of kiwifruit laid a sufficient genetic foundation for protecting and utilizing high-quality resources and selecting excellent breeding material [2].

Among the various *Actinidia* species, *Actinidia eriantha* is a valuable wild species used in kiwifruit breeding programs and is uniquely distributed in various regions of China [14]. Its fruit has extremely high nutritional, medicinal, ornamental, and economic value [15]. The fruit has an extremely high L-ascorbic acid content and possesses various other excellent traits such as high level of phenolic compounds, disease and heat resistance, and long shelf life [16]. In addition, the fruit is easy to peel, and the corolla is pink and has excellent ornamental value [17]. It produces a characteristic berry with great potential after *A. chinensis* and *A. deliciosa* [18]. Thus, the wild *Actinidia eriantha* germplasm has numerous resources, with its excellent fruit properties and wide genetic variations.

Most *A. eriantha* resources grow in the wild or semi-wild environment and differ in fruit quality. Among these, a few varieties are used for developing novel cultivars, and very few are used for commercial production. Moreover, due to the continuous development and irrational exploitation of mountainous areas, coupled with lack of awareness of wild resource protection, the *Actinidia* habitats have been damaged or even predatorily logged, leading to a gradual drop in resources [19]. Therefore, there is an urgent need to protect and utilize germplasm resources. Collection and evaluation of data associated with fruit quality traits can provide a more comprehensive scientific basis for protecting wild resources, facilitating the selection of genetic resources, and promoting the efficient use of elite resources in breeding [20]. In order to utilize the rich *A. eriantha* germplasm resources available in China and to develop high-quality kiwifruit cultivars, our research team has studied kiwifruit resources naturally available in Jiangxi Province [17]. Exploring quality differences and phenotypic diversity of wild *A. eriantha* fruit can provide a theoretical basis for utilizing resources and breeding new kiwifruit.

Based on the previous resource survey, we collected mature fruit samples from a natural wild *A. eriantha* population in Jiangxi Province and analyzed 22 fruit quality traits at the edible stage. Variance, correlation, principal components, and cluster analysis were used to access quality and comprehensive evaluation of the plants. Furthermore, a stepwise regression analysis was used to develop a fruit quality evaluation model based on 236 *A. eriantha* plants. Findings from this study will help establish an efficient system to evaluate the quality of wild *A. eriantha* fruits and to provide a basis for germplasm exploitation and innovation.

## 2. Materials and Methods

### 2.1. Plant and Fruit Material

Wild natural populations of *A. eriantha* were identified in the Luoxiao Mountains of Ganzhou City in the Jiangxi Province, China (E 114°1′1.2″–114°03′92″, N 25°46′24.60″–25°48′86″, altitude 1092.98–1282.79 m). An average SSC of *A. eriantha* fruit reached 6.5% fruit maturity. The strategy for determining the fruit maturity is as follows: from late September to early October 2022, the fruit maturity of these wild populations has been continuously tracked and assessed. Fruits used to detect fruit maturity were collected every 5 days, with 3–5 healthy, nearly mature fruit samples randomly collected from 10–20 bearing plants each time and used to measured the soluble solid content (SSC), until fruit ripening (SSC about 6.5%). Strategies for collecting samples for comprehensive fruit quality evaluation: 20–40 healthy and undamaged mature fruit replicates were harvested from each wild *A. eriantha* plant; a total of 236 wild individual plants of the *A. eriantha* fruits were centrally harvested from wild populations in mid-October 2022. Fruit sampling was carried out from plants located at a distance of at least 50 m. The collected fruits were then transported to the laboratory for processing. Each wild individual plant represented a germplasm, and three fruit samples representing three biological replicates were collected from each germplasm.

### 2.2. Fruit Quality Evaluation

Fruit appearance, internal quality indicators, and antioxidant components were analyzed in this study. The indicators of fruit appearance quality included longitudinal and transverse diameters, fruit shape index, and fruit weight; the quality indicators included dry matter, soluble solids and total sugar contents, titratable acid, solid–acid ratio, sugar–acid ratio, fructose, glucose, sucrose, and quinic, citric, malic, oxalic, lactic, and fumaric acids; the antioxidant components included L-ascorbic acid, total phenols, and total flavonoids.

In total, 8–10 fruits were randomly selected from the fruits for centralized harvest and used to measure the longitudinal and transverse diameter, fruit weight, and dry matter. Fruit weight and dimensions were measured using an electronic balance (Mettler-Toledo Group, Zurich, Swiss Confederation) and digital display vernier calipers (Delixi Electric Power Co., Ltd., Hangzhou, China), respectively. The longitudinal diameter was divided by the transverse diameter to determine the fruit shape index. Dry matter was determined using a 2 mm thick transverse slice cut from the center of the fruit that was dried for 24 h at 65 °C [21] in an electric drying oven (Shanghai Boxun Medical Biological Instrument Corp., Shanghai, China).

The remaining 12–30 fruit replicates were stored in the laboratory at room temperature (25 ± 2 °C) and allowed to ripen until edible (10 N fruit firmness). Fruit firmness was measured using a SMSTA-XT Plus texture analyzer (Stable Micro System Co., Ltd., Surrey, UK) with the following parameters: probe size, 2 mm (diameter) × 24.5 mm (length); drilling, 6 mm; test rate, 1 mm/s. The fruit were rotated 90° after every measurement, and 5 fruit were measured. Subsequently, the soft-ripe fruit were used to measure other quality traits. Total soluble sugar and titratable acid of the fruits were determined following enthrone colorimetry and acid-base titration, respectively [22]. SSC was measured using a PAL-1 hand-held digital display refractometer (Atago Co., Ltd., Tokyo, Japan). Total phenol content was determined using the Folin–Ciocalteu method, while total flavonoid contents were estimated using aluminum nitrate colorimetry; a UV–8000S UV–visible spectrophotometer (Shanghai Metash Instrument Co., Ltd., Shanghai, China) was used to measure the absorbance and determine the levels of these components [23]. Additionally, high-performance liquid chromatography (HPLC) (LC-20A, Shimadzu Corp., Tokyo, Japan) was used to determine the contents of L-ascorbic acid, individual organic acids (quinic, citric, malic, oxalic, lactic, and fumaric), and individual sugars (fructose, glucose, and sucrose). L-ascorbic acid and organic acid were separated using a Shim-pack GISS C18 column at 30 °C. The sulfuric acid solution (0.01 mol·L^−1^, pH 2.6) was used as the mobile phase at a flow rate of 0.5 mL·min^−1^. Detection was achieved at 210 nm [24]. Soluble sugars were separate using a Waters Spherisorb NH_2_ column at 35 °C. An 85% acetonitrile solution was used as the mobile phase at a flow rate of 1.0 mL·min^−1^ [25].

### 2.3. Data Standardization

The membership function method [26] was used to standardize data from 22 quality traits. The positive trait were transformed using Equation (1), and the negative traits were transformed using Equation (2):

Z_ij_ = (Q_ij_ − Q_ijmin_)/(Q_ijmax_ − Q_ijmin_) (1)

Z_ij_ = 1 − (Q_ij_ − Q_ijmin_)/(Q_ijmax_ − Q_ijmin_) (2)

where Z_ij_ is the quality trait (j) membership function value of each germplasm (i), Q_ij_ is the measured value, and Q_ijmax_ and Q_ijmin_ are the maximum and minimum measure values, respectively. The standardized data were used for the subsequent principle components analysis (PCA) and cluster analysis.

### 2.4. Comprehensive Evaluation and Cluster Analysis of Fruit Quality Traits

The Shannon–Wiener information index, representing the genetic diversity index (H’), was calculated according to the method described by Strong et al. [27] to evaluate the diversity in the fruit quality traits. PCA was performed using OriginPro 2024b software (OriginLab Inc., Northampton, MA, USA) to obtain the principal component scores of each sample. The proportion of the contribution rate of the selected principal component to the sum of the contribution rate of the extracted eight principal components was taken as the weight. The product of the scores of the first eight principal components and the corresponding weights of each sample were weighted to obtain the comprehensive score, Dn. This score represented the comprehensive fruit quality of the different *A. eriantha* plants. The systematic cluster analysis based on the 22 fruit traits was performed using the Ward method. Regression analysis was performed using the forward method, with the measure values of 22 quality traits as independent variables.

### 2.5. Statistical Analysis

Microsoft Excel 2019 was used to sort the data and to analyze the maximum, minimum, median, mean, standard deviation, and coefficient of variation. Correlation analyses, PCA, cluster analyses, and regression analyses were performed with the generated data using OriginPro 2024b Statistics (OriginLab Inc., Northampton, MA, USA). Box plots and scatter plots were made with OriginPro 2024b Statistics (OriginLab Inc., Northampton, MA, USA).

## 3. Results

### 3.1. Descriptive Statistics of 22 Fruit Quality Traits

**Figure 1:**
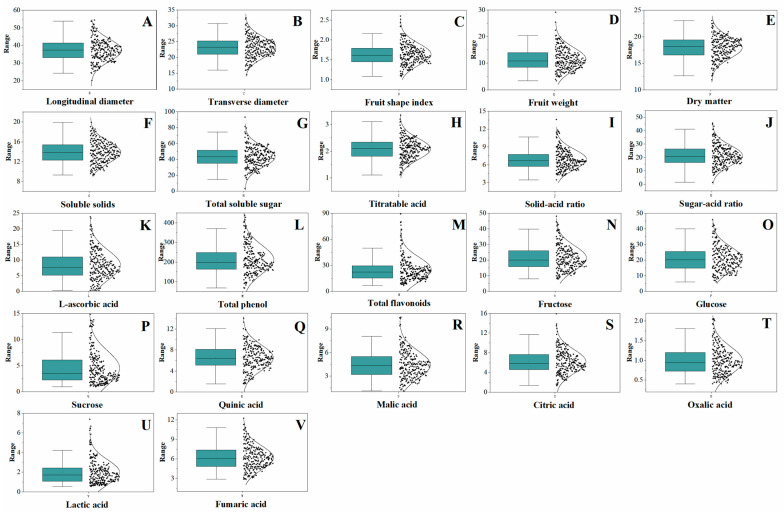
Box plots and scatter plots showing the normal distribution of the fruit quality traits. (**A**) Longitudinal diameter, (**B**) Transverse diameter, (**C**) Fruit shape index, (**D**) Fruit weight, (**E**) Dry matter, (**F**) Soluble solids, (**G**) Total soluble suger, (**H**) TItratable acid, (**I**) Solid-acid ratio, (**J**) Suger-acid ratio, (**K**) L-ascorbic acid, (**L**) Total phenol, (**M**) Total flavonoids, (**N**) Fructose, (**O**) Glucose, (**P**) Surcose, (**Q**) Quinic acid, (**R**) Malic acid, (**S**) Citric acid, (**T**) Oxalic acid, (**V**) Lactic acid, (**U**) Fumaric acid.

The present study analyzed 22 key fruit quality traits on 236 plants of the *A. eriantha* wild natural population, including appearance, internal quality, antioxidant components, soluble sugar components, and organic acids (Table S1). The CV values for the 22 fruit traits ranged from 11.42 to 69.75%, with the highest coefficient for sucrose content and the smallest for dry matter; the average CV was 35.09% (Table 1). Further analysis revealed that genetic diversity index (H’) of these 22 traits ranged from 0.54 to 0.77 (average 0.67), indicating that the fruit quality of the tested samples varied and had large genetic differences. The appearance quality traits, namely longitudinal diameter, transverse diameter, fruit shape index, and fruit weight, exhibited, on average, a CV of 21.83%, with a 14.61–38.44% range. The CV values of the fruit composition-associated traits, including dry matter, SSC, soluble sugars, titratable acid, solid–acid ratio, and sugar–acid ratio, ranged from 11.42 to 36.01%, with an average of 23.05%. The CV values of the antioxidant components, such as L-ascorbic acid, total phenols, and total flavonoids, ranged from 35.14 to 58.32%, with an average of 50.01%. CV values for the soluble sugar components, including fructose, glucose, and sucrose, ranged from 38.02 to 69.75%, with an average of 48.95%. The average CV observed for the organic acids, such as quinic, citric, malic, oxalic, lactic, and fumaric, was 41.58% (30.26–64.95% range). Thus, this study found that the CV values of the different quality traits varied, and the CV range observed for the antioxidant components, soluble sugars, and organic acids was significantly greater than that for the appearance and intrinsic quality traits. Additionally, according to the kurtosis and skewness presented in Table 1 and the box plots and scatter plots, the data displayed a near-normal distribution regarding quality traits (Figure 1).

### 3.2. Correlation Clustering of 22 Fruit Quality Traits

**Figure 2:**
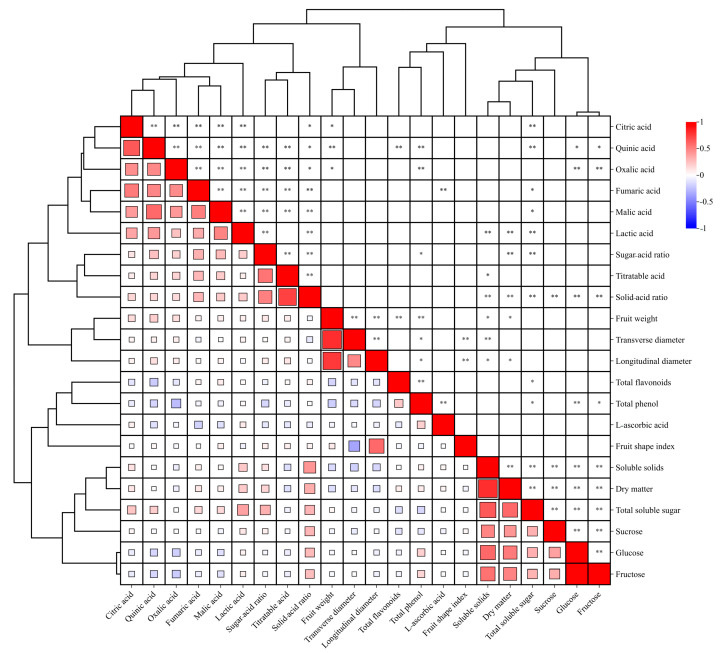
**Correlation clustering heat map of the 22 fruit quality traits.** Positive correlations are shown in red and negative correlations are shown in blue. Here, * and ** represent statistical significance at *p* < 0.05 and *p* < 0.01, respectively.

Furthermore, this study found strong correlations among different quality traits (Figure 2). Sugar-related traits, such as fructose, glucose, sucrose, total soluble sugar, fruit dry matter, and soluble solids, were positively correlated with one another. Subsequent clustering analysis based on the correlation coefficients grouped these 22 quality traits according to type. For instance, dry matter, soluble solids, total soluble sugar, fructose, glucose, and sucrose showed significant positive correlations with each other and clustered in one category (*p* < 0.01). Appearance-, antioxidant-, and acid-related traits also exhibited similar results. These observations indicated that a few traits provided the same information. Therefore, the overlapping traits were avoided to simplify the evaluation.

### 3.3. Principal Component Analysis of the Fruit Quality Traits

Furthermore, the Kaiser–Meyer–Olkin (KMO) test and the Bartlett test were performed. Typically, when the KMO test value is greater than 0.5 and the Bartlett sphericity test value is less than 0.01, the correlation between the variables is considered strong, and the variables are considered suitable for PCA. In this study, the KMO test value was 0.672, and the Bartlett sphericity test *p*-value was less than 0.01, indicating the suitability of this dataset for PCA. A total of eight principal components with eigenvalues greater than one were extracted; their cumulative contribution rate was 77.93% (Table 2).

In PCA, the eigenvalue reflects each variable’s importance in the principal component; the greater the absolute load value, the more significant the impact of the trait on the principal component. The contribution rate of the first principal component (PC1) was 19.00%, and the representative traits of this component included dry matter, soluble solids, total soluble sugar, and solid–acid ratio, which mainly reflected the sweetness of the fruit. Similarly, a 17.39% contribution rate was detected for the second principal component (PC2), which included fructose, glucose, and quinic acid, reflecting the sweetness and sourness of the fruits. The contribution rate of the third principal component (PC3) was 10.80%, and longitudinal diameter, transverse diameter, and fruit weight, primarily reflecting the fruit’s appearance quality, were this component’s representative traits. The contribution rate of the fourth principal component (PC4) was 8.37%, and the representative trait was titratable acid, reflecting the sourness of the fruit. The contribution rate of the fifth principal component (PC5) was 6.57%, and the representative trait was the fruit shape index, mainly reflecting the appearance of the fruit. The contribution rates of the sixth (PC6), seventh (PC7), and eighth (PC8) principal components were 6.04%, 5.10%, and 4.66%, respectively, and the representative traits of these components were total phenols, L-ascorbic acid, and total flavonoids, primarily reflecting the nutritional characteristics of the fruit.

Subsequently, the comprehensive evaluation score, Dn, was calculated by multiplying the contribution rate of each component (the weight of each principal component). The Dn value obtained was equal to 0.24F1 + 0.22F2 + 0.14F3 + 0.11F4 + 0.08F5 + 0.08F6 + 0.06F7 + 0.06F8. Then, the plants were ranked based on the Dn values. The higher the Dn, the better the comprehensive quality of the fruit. The obtained comprehensive score and the rank of the 236 *A. eriantha* wild plants are shown in Table 3. Among the 236 plants, CSA31, CSA243, CSA11, CSA157, and CSA248 were ranked in the top 5 (Dn > 1.04), indicating excellent fruit quality. On the contrary, CSA134, CSA37, CSA168, CSA190, and CSA70 were the bottom five plants (Dn < −0.8), indicating poor fruit quality (Table 3).

### 3.4. Cluster Analysis

**Figure 3:**
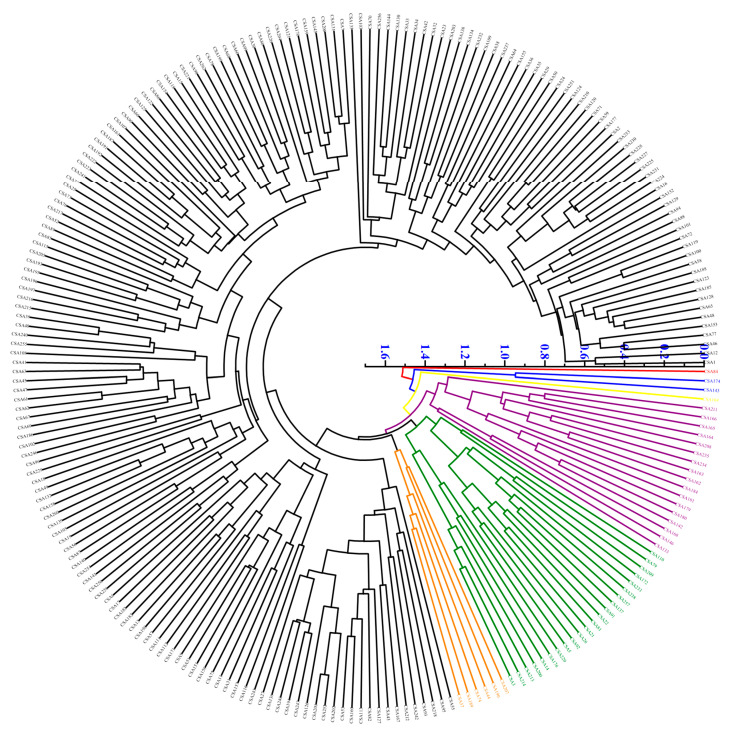
**Cluster analysis of 236 wild *A. eriantha* germplasm plants.** Category I are shown in red, category II are shown in blue, category III are shown in yellow, category IV are shown in purple, category V are shown in green, category VI are shown in orange, and category VII are shown in blank.

Furthermore, a systematic cluster analysis was performed to assess the quality characteristics and genetic relationships among the 236 wild *A. eriantha* plants. The analysis showed that CSA84 separated from all samples at a squared Euclidean distance of 1.5 (Figure 3). CSA84 formed a separate category because of its extremely high level of L-ascorbic acid and high levels of dry matter, soluble solids, total sugar, fructose, and glucose. At a square Euclidean distance 1.35, the 236 *A. eriantha* samples were subdivided into seven categories. Category I contained one germplasm (CSA84) with abundant fruit substances and strong fruit flavor. Category II contained two germplasms (CSA174 and CSA143), which had high fructose, glucose, sugar–acid ratio, and citric acid. Category III contained one germplasm (CSA104), with low dry matter, soluble solids, soluble sugar levels, and low solid–acid and sugar–acid ratios but high quinic acid and malic acid levels, suggesting low quality. Consistent with this observation, the rank of CSA104, based on the comprehensive evaluation of PCA, was 190, which indicated poor flavor quality. Category IV contained 17 germplasms (CSA211, CSA166, CSA165, CSA164, CSA208, CSA233, CSA234, CSA163, CSA162, CSA184, CSA181, CSA179, CSA180, CSA142, CSA168, CSA146, and CSA133), which had high content of dry matter, fructose, glucose, total flavonoids, and total phenols. Category V included 22 germplasms (CSA110, CSA79, CSA209, CSA172, CSA233, CSA238, CSA257, CSA137, CSA91, CSA22, CSA81, CSA21, CSA26, CSA92, CSA5, CSA220, CSA176, CSA14, CSA200, CSA213, CSA214, and CSA3); the fruit of all these germplasms was long, with high dry matter and sucrose level and low phenol content. Category VI contained six germplasms (CSA207, CSA190, CSA44, CSA74, CSA189, and CSA37); this category corresponded to a small phenotype with low dry matter and soluble solids and high acidity. The overall fruit quality was low in these plants, which is consistent with the results based on the comprehensive evaluation. The remaining 187 accessions belonged to category VII, accounting for 79.24% of the germplasm. Overall, the germplasms clustered in the same category had the same quality characteristics.

### 3.5. Regression Modeling and Predictive Capacity of the Traits

The regression model was as follows, Y = −2.13 + 0.64X1 + 1.24X2 + 0.93X3 + 0.71X4 + 0.71X5 + 0.18X6 + 0.22X7 + 0.24X8 + 0.19X9, where X1 to X9 represent sugar–acid ratio, fruit weight, fruit shape index, malic acid, solid–acid ratio, citric acid, L-ascorbic acid, quinic acid, and total soluble sugar, respectively (Table 4). The adjusted *R*^2^ value was 0.945, and the *p*-value was <0.0001, indicating the consistency of the model with the comprehensive evaluation in effectively predicting and evaluating the fruit quality. The model covered more quality indicators associated with appearance, sweetness, sourness, flavor, and nutritional value. In addition, these nine key traits selected based on stepwise regression showed high consistency with the representative indicators extracted based on the principal components, confirming that the nine traits could be used to evaluate *A. eriantha* plants comprehensively.

## 4. Discussion

Elite germplasm resources are selected for breeding primarily based on a comprehensive evaluation. Therefore, the traits used for evaluation are critical; the selected traits should represent the nutritional and quality characteristics of the fruit. Moreover, the evaluation system should be based on sufficient samples and indicators. The present study analyzed 22 fruit quality traits, including appearance, flavor, and nutritional indicators, from 236 natural populations. In other words, this study adopted a large sample size and different quality indicators, meeting the two basic requirements of comprehensive evaluation [28]. Therefore, this system is highly representative and lays a foundation for accurately evaluating the quality of fruit produced by *A. eriantha* plants. The findings based on this system will eventually accelerate resource mining, breeding novel varieties, and constructing a core germplasm.

Rich germplasm resources provide a solid material basis for breeding novel varieties and conducting biotechnology research in plants, including kiwifruit [1]. Investigating and analyzing the major fruit quality traits will directly reveal the differences between individual plants and provide a theoretical basis for resource conservation, specific germplasm exploration, and breeding. In this study, an analysis of the edible fruit samples revealed extensive variation in the quality traits of 236 wild *A. eriantha* plants in a natural environment. Similar observations have been reported on other wild populations and main cultivars of *A. eriantha* in different regions [18]. The difference in CV indirectly reflects the difference in evolutionary conservation and genetic plasticity, which are essential for evaluating and screening resources [29]. The greater the CV of the measured traits, the higher the genetic background and the contribution rate to germplasm innovation, which is more conducive to the application of germplasm resources [30,31]. Thus, the CV in 22 fruit quality traits confirms a high variation level and availability of a rich *A. eriantha* germplasm for developing novel kiwifruit varieties. Notably, the variation in internal quality was greater than that in the appearance quality. The CV in the fruit shape index, soluble solids, and titratable acid was small and indicated high genetic stability, suggesting that these quality traits were less affected by geographical and environmental factors. However, a large degree of variation was detected in single fruit weight, total soluble sugar, solid–acid ratio, sucrose, malic acid, total phenol, and total flavonoids, indicating rich genetic diversity. Earlier studies reported that the CV values for L-ascorbic acid for the natural populations of Magu Mountain and Wugong Mountain were 19.50% [32] and 21.88% [33], respectively, and that for the cultivated varieties were 30.87% (in *A. chinensis*) and 37.88% (in *A. deliciosa*) [17,34]. These values are significantly lower than the CV of the germplasm tested in this study. However, the CV for L-ascorbic acid of *A. arguta* (56.58%) [27] is equivalent to the degree of variation in the tested germplasm (53.08%). These high CV values indicate high variation in L-ascorbic acid in different *Actinidia* species, possibly due to the influence of environment, species, and level of cultivation management [35].

Furthermore, the correlation clustering heat map indicated different degrees of correlation between the traits. Notably, the same attributes or the same type of quality traits were strongly correlated, suggesting an overlap in the information provided by a few traits. Therefore, the redundant indicators were removed initially, simplifying the evaluation process. PCA and cluster analysis have been adopted to narrow down the traits and comprehensively evaluate the germplasms of several plant species. Assessment of germplasm resources of tomato [36], raspberry [37], wolfberry [38], camellia [39], sorghum [40], and various other crops using these two methods have guided us in the efficient production and screening of germplasm. Therefore, this study comprehensively evaluated the fruit quality traits using PCA and cluster analysis. By means of PCA, the 22 quality traits provided eight new, comprehensive, and independent principal components, covering 77.93% of the information on the quality traits of the fruits. These eight PCs could reflect the overall quality traits of fruits from the plants. Among the eight PCs, the one with the largest contribution rate included soluble solids, total soluble sugar, solid–acid ratio, and sugar–acid ratio, indicating the significant impact of these four traits on *A. eriantha* fruit quality. Interestingly, these four traits are also known to greatly influence fruit quality in *A. chinensis* and *A. deliciosa* [34]. Of these, soluble solids and total soluble sugar content are related to fruit sweetness, while the solid–acid ratio and sugar–acid ratio reflect fruit flavor [41]. The second PC identified in this study mainly included the quinic acid, which means sourness also considerably influences fruit flavor. The third PC included the fruit appearance traits (weight, longitudinal diameter, transverse diameter). These observations based on PCA are consistent with the reports on the yellow-fleshed varieties and the major cultivars of kiwifruit in different producing areas. In addition, *A. eriantha* fruit is well-known for its extremely high L-ascorbic acid content [42]. However, in *A. eriantha*, L-ascorbic acid content appeared in the PC with a small contribution rate. This observation is in contrast to the highest contribution rate of L-ascorbic acid in cultivated kiwifruit cultivars. This difference is probably due to the high genetic variation and heritability of the *A. eriantha* population [43,44]. Still, based on the earlier reports, we suggest that L-ascorbic acid should be considered a typical fruit feature and an essential evaluation trait for *A. eriantha*.

Furthermore, this study grouped the 236 germplasms into seven categories using a cluster analysis. The germplasms of each category had characteristics different from those in other categories. This grouping provides a scientific basis for selecting and utilizing parental materials with specific traits for targeted breeding. Finally, combining the observations obtained from PC comprehensive evaluation and cluster analysis, the germplasms were screened based on their traits: high sugar and high L-ascorbic acid (such as CSA84), high sugar and high organic acid (CSA104), high sugar and high phenolic acid (CSA211, CSA166, CSA165, CSA164, CSA208, CSA233, CSA234, CSA163, CSA162, CSA184, CSA181, CSA179, CSA180, CSA142, CSA168, CSA146, and CSA133), large fruit (CSA243, CSA198, CSA116, and CSA57). These findings provide a reference for researchers and breeders to carry out directional breeding.

The comprehensive evaluation of germplasm resources, combining the membership function method with PCA, helped simplify resource screening and reflected the excellent traits [45]. In this study, a stepwise regression analysis was performed on these 22 fruit quality traits, and a quality prediction model with a high fitting level was generated. The fitting degree between the comprehensive evaluation score of 236 *A. eriantha* plants obtained using the prediction model and the comprehensive score of PCA in linear regression was as high as 0.945. In addition, nine key evaluation traits were screened based on the stepwise regression analysis. These traits are similar to the those extracted using PCA, representing the appearance, sweetness, sourness, flavor, and nutritional attributes of *A. eriantha* [34]. Thus, the study proposes that these nine traits are efficient indicators to evaluate *A. eriantha* comprehensively, facilitating their efficient utilization. Therefore, future works on *A. eriantha* evaluation should focus on these phenotypic traits.

## 5. Conclusions

This study, based on the analysis of 22 quality traits of 236 wild *A. eriantha* plants, proved their phenotypic and genetic diversity and indicated that these wild *Actinidia* plants of China serve as potential breeding resources. Furthermore, a high-quality prediction model with high fitting was obtained using these data, and nine key evaluation traits proved to be effective in evaluating germplasm resources. Overall, this study establishes a model for predicting the quality of *A. eriantha* fruits, identifies representative evaluation traits, and constructs a fruit quality evaluation system. These findings will promote the domestication and cultivation of the wild *A. eriantha* resources.

## Figures and Tables

**Table 1:** Descriptive statistics of 22 fruit quality traits of 236 wild *A. eriantha* plants.

Quality Traits	Minimum	Maximum	Median	Mean	SD	CV	H’	Skewness	Kurtosis
Longitudinal diameter (mm)	20.21	54.40	37.38	37.38	6.40	17.12	0.72	0.29	0.06
Transverse diameter (mm)	14.52	36.25	23.19	23.21	3.39	14.61	0.70	0.31	0.87
Fruit shape index	1.08	2.82	1.64	1.62	0.28	17.16	0.69	0.90	1.90
Fruit weight (g)	3.39	32.45	11.64	10.82	4.47	38.44	0.75	1.14	2.86
Dry matter (%)	11.88	23.06	17.97	18.18	2.05	11.42	0.65	−0.28	−0.11
Soluble solids (%)	9.30	19.92	13.93	13.90	2.19	15.75	0.63	0.16	−0.41
Total soluble sugar (%)	3.32	93.05	43.41	43.32	13.47	31.03	0.62	0.27	0.77
Titratable acid (%)	1.11	3.36	2.09	2.10	0.40	19.21	0.62	0.16	0.24
Solid–acid ratio	3.45	13.61	6.92	6.66	1.72	24.90	0.70	0.89	1.16
Sugar–acid ratio	1.44	45.68	21.53	20.75	7.75	36.01	0.68	0.51	0.40
L-ascorbic acid (mg/g)	0.28	23.86	8.47	7.80	4.79	56.58	0.63	0.73	0.54
Total phenols (mg/100 g)	67.89	438.93	213.13	198.31	74.89	35.14	0.61	0.77	0.32
Total flavonoids (mg/100 g)	6.56	89.18	25.30	22.27	14.75	58.32	0.54	1.86	4.34
Fructose (mg/g)	8.01	48.15	21.40	20.05	8.13	38.02	0.75	0.80	0.52
Glucose (mg/g)	5.98	45.84	20.71	20.06	8.09	39.09	0.75	0.65	0.26
Sucrose (mg/g)	0.98	15.19	4.61	3.54	3.22	69.75	0.77	1.39	1.39
Quinic acid (mg/g)	1.56	15.31	6.56	6.43	2.50	38.10	0.63	0.47	1.03
Citric acid (mg/g)	1.35	15.93	6.25	5.90	2.49	39.82	0.63	0.79	0.90
Malic acid (mg/g)	1.12	10.53	4.46	4.34	1.79	40.19	0.65	0.67	1.01
Oxalic acid (mg/g)	0.41	2.06	1.00	0.96	0.36	36.14	0.60	0.80	0.50
Lactic acid (mg/g)	0.55	7.39	2.01	1.71	1.30	64.95	0.72	1.63	3.15
Fumaric acid (mg/100 g)	2.89	12.26	6.21	6.08	1.88	30.26	0.61	0.57	0.19
Mean	-	-	-	-	-	35.09	0.67	0.70	1.00

**Table 2:** Eigenvalues and contribution rates of eight principle components.

	PC1	PC2	PC3	PC4	PC5	PC6	PC7	PC8
Longitudinal diameter	−0.02	0.38	0.77	0.16	0.37	0.24	−0.05	0.07
Transverse diameter	−0.06	0.30	0.72	−0.17	−0.55	0.07	−0.01	0.11
Fruit shape index	0.02	0.10	0.18	0.32	0.89	0.21	0.00	0.00
Fruit weight	0.04	0.43	0.84	−0.07	−0.13	0.15	−0.01	0.08
Dry matter	0.65	−0.51	0.05	−0.12	0.04	0.01	−0.04	0.25
Soluble solids	0.69	−0.57	0.05	−0.10	0.10	−0.05	−0.01	0.15
Total soluble sugar	0.69	−0.22	0.12	−0.19	0.08	−0.24	0.09	0.37
Titratable acid	0.30	0.36	−0.06	0.76	−0.25	0.01	0.18	−0.13
Solid–acid ratio	0.67	−0.01	−0.05	0.60	−0.16	0.02	0.14	−0.08
Sugar–acid ratio	0.50	0.27	−0.02	0.49	−0.11	−0.17	0.17	0.25
L-ascorbic acid	−0.08	−0.13	−0.01	−0.21	0.06	0.21	0.85	0.05
Total phenol	−0.09	−0.31	−0.23	0.00	−0.11	0.71	0.17	−0.11
Total flavonoids	−0.07	−0.11	−0.30	0.18	−0.13	0.49	−0.39	0.45
Fructose	0.43	−0.65	0.29	0.01	−0.10	0.20	−0.12	−0.37
Glucose	0.45	−0.67	0.30	0.01	−0.09	0.19	−0.10	−0.36
Sucrose	0.44	−0.36	0.17	0.01	0.14	−0.29	−0.07	−0.08
Quinic acid	0.49	0.61	−0.09	−0.27	0.08	0.00	−0.02	−0.17
Malic acid	0.49	0.50	−0.23	−0.12	0.04	0.24	−0.17	0.07
Citric acid	0.50	0.45	−0.12	−0.39	0.07	0.09	0.10	−0.20
Oxalic acid	0.33	0.57	−0.12	−0.17	0.01	−0.13	−0.04	−0.25
Lactic acid	0.55	0.22	−0.11	−0.30	−0.05	0.24	0.13	0.24
Fumaric acid	0.53	0.47	−0.25	−0.03	−0.04	0.16	−0.19	−0.14
Eigenvalue	4.18	3.83	2.38	1.84	1.45	1.33	1.12	1.03
Contribution rates	19.00	17.39	10.80	8.37	6.57	6.04	5.10	4.66
Cumulative contribution rates	19.00	36.38	47.19	55.56	62.13	68.17	73.27	77.93

**Table 3:** Comprehensive score and rank of 236 wild *A. eriantha* plants.

	Score	Rank		Score	Rank		Score	Rank		Score	Rank		Score	Rank
CSA1	−0.2	159	CSA55	0.09	88	CSA105	−0.37	194	CSA160	0.13	79	CSA212	0.15	71
CSA2	−0.39	198	CSA56	0.1	85	CSA107	−0.13	147	CSA161	−0.24	170	CSA213	0.45	32
CSA3	0.21	64	CSA57	0.19	66	CSA108	−0.33	189	CSA162	0.23	61	CSA214	0.46	29
CSA5	0.11	84	CSA58	−0.4	202	CSA109	−0.44	210	CSA163	0.49	26	CSA215	0.05	98
CSA6	−0.52	223	CSA59	−0.49	219	CSA110	0.54	21	CSA164	−0.16	156	CSA216	0.09	89
CSA7	0.23	60	CSA60	0.17	70	CSA111	−0.39	197	CSA165	−0.24	169	CSA217	−0.08	134
CSA9	0.93	6	CSA61	0.24	55	CSA112	0.04	101	CSA166	−0.26	176	CSA218	0.15	75
CSA11	1.11	3	CSA62	0.08	91	CSA113	−0.21	162	CSA167	0.27	49	CSA220	0.01	111
CSA12	−0.02	121	CSA63	−0.11	140	CSA115	0.25	54	CSA168	−0.87	234	CSA221	0.12	81
CSA13	0.46	31	CSA64	0.32	44	CSA116	0.51	23	CSA170	−0.09	138	CSA222	−0.28	181
CSA14	0.24	57	CSA65	0.15	74	CSA118	0.07	93	CSA171	0.01	110	CSA223	−0.26	179
CSA15	−0.07	132	CSA66	−0.14	150	CSA119	0.35	39	CSA172	−0.4	201	CSA224	−0.07	131
CSA16	−0.14	151	CSA67	−0.52	222	CSA120	−0.23	165	CSA174	0.27	48	CSA225	−0.41	204
CSA17	0.18	67	CSA68	0.09	87	CSA121	−0.11	142	CSA176	−0.36	193	CSA226	−0.16	154
CSA18	−0.23	168	CSA69	−0.48	218	CSA122	0.05	97	CSA177	−0.47	214	CSA227	−0.26	175
CSA19	−0.14	148	CSA70	−1.05	236	CSA123	0.29	47	CSA179	−0.43	208	CSA228	−0.38	195
CSA21	0.14	78	CSA71	−0.03	123	CSA124	−0.01	117	CSA180	−0.23	166	CSA229	−0.61	227
CSA22	0.26	53	CSA72	0.09	90	CSA125	0	113	CSA181	0.17	69	CSA230	−0.45	212
CSA23	−0.24	171	CSA73	−0.12	144	CSA126	0.87	9	CSA182	−0.08	133	CSA231	−0.07	130
CSA24	−0.05	124	CSA74	−0.59	226	CSA127	0.34	40	CSA183	0.07	94	CSA232	−0.79	230
CSA25	−0.26	177	CSA75	0.5	25	CSA128	0	114	CSA184	0.04	102	CSA233	0.03	105
CSA26	0.67	14	CSA76	−0.13	146	CSA129	0.33	43	CSA185	0.22	62	CSA234	0	112
CSA27	0.92	7	CSA77	−0.31	187	CSA130	−0.1	139	CSA186	0.09	86	CSA235	0.43	33
CSA29	0.19	65	CSA78	−0.21	164	CSA131	0.46	30	CSA187	−0.48	216	CSA237	−0.26	174
CSA30	0.14	76	CSA79	0.39	34	CSA132	0.03	104	CSA188	−0.02	122	CSA238	−0.07	129
CSA31	1.42	1	CSA80	0.17	68	CSA133	0.05	100	CSA189	−0.76	229	CSA239	−0.01	118
CSA32	−0.41	205	CSA81	0.27	51	CSA134	−0.8	232	CSA190	−0.99	235	CSA240	−0.53	224
CSA33	−0.01	115	CSA82	0.06	96	CSA135	−0.26	178	CSA191	−0.48	217	CSA241	−0.35	192
CSA34	−0.2	160	CSA84	0.65	16	CSA136	0.64	17	CSA192	−0.35	191	CSA242	0.46	28
CSA35	0.23	59	CSA85	0.23	58	CSA137	0.27	50	CSA193	−0.09	137	CSA243	1.14	2
CSA36	0.57	20	CSA86	0.05	99	CSA138	−0.43	207	CSA194	0.77	10	CSA246	−0.44	211
CSA37	−0.85	233	CSA87	0.3	46	CSA139	−0.21	163	CSA195	−0.13	145	CSA247	0.9	8
CSA38	−0.62	228	CSA88	0.51	24	CSA140	0.01	109	CSA196	−0.39	199	CSA248	1.04	5
CSA39	−0.31	186	CSA89	−0.02	120	CSA141	−0.3	185	CSA197	0.22	63	CSA250	−0.32	188
CSA40	−0.16	155	CSA90	−0.16	153	CSA142	0.14	77	CSA198	0.6	19	CSA251	0.15	72
CSA41	0.03	107	CSA91	0.26	52	CSA143	−0.05	126	CSA199	−0.05	125	CSA252	0.74	11
CSA42	−0.47	215	CSA92	0.66	15	CSA144	−0.26	180	CSA200	0.31	45	CSA253	−0.09	136
CSA43	0.37	36	CSA93	0.03	103	CSA145	0.07	92	CSA201	0.69	13	CSA254	−0.29	183
CSA44	−0.5	220	CSA94	0.11	83	CSA146	−0.79	231	CSA202	0.34	42	CSA255	−0.09	135
CSA45	−0.28	182	CSA95	0.38	35	CSA151	0.48	27	CSA203	−0.51	221	CSA256	−0.46	213
CSA46	−0.2	161	CSA96	−0.23	167	CSA152	0.37	37	CSA204	−0.19	158	CSA257	0.15	73
CSA47	−0.12	143	CSA98	0.11	82	CSA153	−0.3	184	CSA205	0.03	106	CSA258	−0.39	200
CSA48	−0.17	157	CSA99	0.07	95	CSA154	−0.14	149	CSA206	0.34	41	CSA260	−0.25	172
CSA49	−0.01	119	CSA100	0.35	38	CSA155	0.61	18	CSA207	−0.41	206	CSA262	−0.11	141
CSA50	−0.06	128	CSA101	−0.14	152	CSA156	0.69	12	CSA208	0.24	56			
CSA51	0.54	22	CSA102	−0.41	203	CSA157	1.11	4	CSA209	−0.38	196			
CSA52	0.03	108	CSA103	−0.25	173	CSA158	−0.01	116	CSA210	−0.44	209			
CSA53	−0.06	127	CSA104	−0.34	190	CSA159	0.12	80	CSA211	−0.57	225			

**Table 4:** Basic parameters of the model for predicting *A. eriantha* fruit quality.

Model	Non-Standardized Coefficient		Standardized Coefficient	t	Significance
	B	Standard Error	Beta		
Constant	−2.13	0.04	-	−47.77	0.00
X1 (Sugar–acid ratio)	0.64	0.04	0.27	15.87	0.00
X2 (Fruit weight)	1.24	0.04	0.45	31.19	0.00
X3 (Fruit shape index)	0.93	0.04	0.36	25.56	0.00
X4 (Malic acid)	0.71	0.06	0.21	11.91	0.00
X5 (Solid–acid ratio)	0.71	0.04	0.29	17.59	0.00
X6 (Citric acid)	0.18	0.03	0.12	6.00	0.00
X7 (L-ascorbic acid)	0.22	0.03	0.11	7.47	0.00
X8 (Quinic acid)	0.24	0.05	0.11	4.95	0.00
X9 (Total soluble sugar)	0.19	0.04	0.07	4.46	0.00

## Data Availability

The data presented in this study are available on request from the corresponding authors. The data are not publicly available due to privacy restrictions.

## Supplementary Materials

The following supporting information can be downloaded at: https://www.mdpi.com/article/10.3390/foods13244014/s1, Table S1: Supplement material-Raw data of fruit quality.

## References

[1] Xu X.B., Zhang Q.M. (2003). Researches and utilizations of germplasm resources of kiwifruit in China. Chin. Bull. Bot..

[2] Zhong C.H., Huang W.J., Li D.W., Zhang Q., Li L. (2021). Dynamic analysis of kiwifruit industry development and fresh fruit trade in the world. China Fruits.

[3] Julia B.S., Jane E.P., Elizabeth A.A., Giles E.D., Julian I.S. (2019). Genetic strategies for improving crop yields. Nature.

[4] Huang H.W., Liu Y.F. (2014). Natural hybridization, introgression breeding, and cultivar improvement in the genus *Actnidia*. Tree Genet. Genomes.

[5] Huang H.W., Wang S.M., Jiang Z.W., Zhang Z.H., Huang R.H., Cheng Z.P., Gong J.J., Chen X.Z. (2005). Yellow-flesh kiwifruit cultivar ‘Jintao’. Acta Hortic. Sin..

[6] Huang H.W., Jiang Z.W., Zhong C.H., Zhang Z.H., Wang S.M., Huang R.H. (2008). Tetraploid and high-quality kiwifruit cultivar ‘Wuzhi3’. Acta Hortic. Sin..

[7] Wang S.M., Zhang Z.H., Jiang Z.W., Huang H.W., Huang R.H., Cheng Z.P., Gong J.J., Chen X.Z. (2004). A new kiwifruit variety ‘Jinxia’. Acta Hortic. Sin..

[8] Huang H.W., Zhang Z.H., Jiang Z.W., Cheng Z.P., Gong J.J., Chen X.Z., Wang S.M. (2005). A new extra early ripening kiwifruit variety ‘Jinzao’. Acta Hortic. Sin..

[9] Huang Y.L. (1990). Lushanxiang-Actinidia chinensis new variety. Plants.

[10] Huang H.W., Zhang Z.H., Jiang Z.W., Cheng Z.P., Chen X.Z., Wang Y.C., Gong J.J., Wang S.M. (2006). A new male cultivar of *Actinidia chinensis* ‘Moshan 4’. Acta Hortic. Sin..

[11] Zhang H.Q., Yao X.R., Lu L.H., Gu X.B., Song G.H., Xie M. (2023). Breeding of a kiwifruit cultivar Sweet White (*Actinidia eriantha*). J. Fruit Sci..

[12] Liao G.L., Huang C.H., Jia D.F., Zhong M., Tao J.J., Qu X.Y., Xu X.B. (2023). A high-quality genome of *Actinidia eriantha* provides new insight into ascorbic acid regulation. J. Integr. Agric..

[13] Zhong C.H., Li D.W., Zhang Q., Li L., Huang W.J., Han F. Identification, creation and utilization of the germplasm resources of kiwifruit. Proceedings of the III International Symposium on Fruit Culture along Silk Road Countries.

[14] Liao G.L., Xu X.B., Huang C.H., Zhong M., Jia D.F. (2021). Resource evaluation and novel germplasm mining of *Actinidia eriantha*. Sci. Hortic..

[15] Richardson D.P., Ansell J., Drummond L.N. (2018). The nutritional and health attributes of kiwifruit: A review. Eur. J. Nutr..

[16] Tao J.J., Jia H.M., Wu M.T., Zhong W.Q., Huang Y.Q., Huang L.H., Xu Y., Huang C.H. (2024). Integrated metabolome and transcriptome analysis reveals the mechanism related to the formation of peelability in *Actinidia eriantha*. Sci. Hortic..

[17] Ross G.A., Neelam N.S., Ian C.H., Sarah L.J., Roswitha S. (2009). *Actinidia eriantha*: A parental species for breeding kiwifruit with novel peelability and health attributes. N. Z. J. For. Sci..

[18] Xu X.B., Liao G.L., Huang C.H., Zhong M. (2021). Germplasm Resources of Actinidia eriantha.

[19] Yu W.H., Wu B.F., Wang X.Y., Yao Z., Li Y.H., Liu Y.B. (2020). Scale-dependent effects of habitat fragmentation on the genetic diversity of *Actinidia chinensis* populations in China. Hortic. Res..

[20] Verma H., Borah J.L., Sarma R.N. (2019). Variability assessment for root and drought tolerance traits and genetic diversity analysis of rice germplasm using SSR markers. Sci. Rep..

[21] Wu J.H. (2015). Pollen effects on fruit attributes and seed properties in colchicine-induced autotetraploids of red-fleshed kiwifruit (*Actinidia chinensis* Planch.). J. Hortic. Sci. Biotechnol..

[22] Brito A.A., Campos F., Reis N.A., Damiani C., Silva F.A., Almeida T.G., Júnior L.C. (2022). Non-destructive determination of color, titratable acidity, and dry matter in intact tomatoes using a portable Vis-NIR spectrometer. J. Food Compos. Anal..

[23] Leontowicz H., Leontowicz M., Latocha P., Jesion I., Park Y.S., Katrich E., Barasch D., Nemirovski A., Gorinstein S. (2016). Bioactivity and nutritional properties of hardy kiwi fruit *Actinidia arguta* in comparison with *Actinidia deliciosa* ‘Hayward’and *Actinidia eriantha* ‘Bidan’. Food Chem..

[24] Wang L.R., Li R., Shi X.J., Wei L., Li W., Shao Y. (2023). Ripening patterns (off-tree and on-tree) affect physiology, quality, and ascorbic acid metabolism of mango fruit (cv. Guifei). Sci. Hortic..

[25] Kim D., Son J.E. (2022). Adding far-red to red, blue supplemental light-emitting diode interlighting improved sweet pepper yield but attenuated carotenoid content. Front. Plant Sci..

[26] Jain A., Sharma A. (2020). Membership function formulation methods for fuzzy logic systems: A comprehensive review. J. Crit. Rev..

[27] Strong W.L. (2016). Biased richness and evenness relationships within Shannon–Wiener index values. Ecol. Indic..

[28] Wen J.L., Cao W.Y., Wang Y., He Y.L., Sun Y.N., Yuan P.Q., Sun B.W., LU W.P. (2024). Comprehensive evaluation of fruit quality of *Actinidia arguta* based on principal component analysis and cluster analysis. Sci. Technol. Food Ind..

[29] Saballos A., Williams I.M. (2024). Genome-wide association study identifies candidate loci with major contributions to the genetic control of pod morphological traits in snap bean. J. Am. Soc. Hortic. Sci..

[30] Alizadeh K., Fatholahi S., Silva J.A.T.d. (2015). Variation in the fruit characteristics of local pear (*Pyrus* spp.) in the northwest of Iran. Genet. Resour. Crop Evol..

[31] Wu F.Z., Zhang R.M., Yin Z.Y., Wang H.X. (2022). Comprehensive quality evaluation of highbush blueberry cultivars based on principal component analysis. Trans. Chin. Soc. Agric. Eng..

[32] He Y.Q., Li Z.Y., Liao G.L., Chen L., Zhong M., Huang C.H., Jia D.F., Qu X.Y., Xu X.B. (2020). Variation in fruit quality within wild *Actinidia eriantha* germplasm. N. Z. J. Crop Hortic. Sci..

[33] Tang J.L., Huang C.H., Liu K.P., Gao J., Wu H., Xu X.B. (2013). A mutation analysis of ascorbic acid content in the leaves and fruit of wild *Actinidia eriantha* Benth. Acta Agric. Univ. Jiangxiensis.

[34] Guo L.L., Pang R.L., Wang R.P., Qiao C.K., Tian F.J., Wang C.X., Li J., Pang T., Cheng X., Xie H.Z. (2022). Comprehensive trait evaluation for kiwifruit nutritional quality. J. Fruit Sci..

[35] Castro J.C., Castro C.G., Cobos M. (2023). Genetic and biochemical strategies for regulation of L-ascorbic acid biosynthesis in plants through the L-galactose pathway. Front. Plant Sci..

[36] Chang Y.L., Zhang X.D., Wang C., Ma N., Xie J.M., Zhang J. (2024). Fruit quality analysis and flavor comprehensive evaluation of cherry tomatoes of different colors. Foods.

[37] Yu Y.P., Yang G., Sun L.Y., Song X.S., Bao Y.H., Luo T., Wang J.L. (2022). Comprehensive evaluation of 24 red raspberry varieties in Northeast China based on nutrition and taste. Foods.

[38] Ma Z.H., Yin J., Yang Y.P., Sun F.B., Yang Z. (2023). Effect of water and nitrogen coupling regulation on the growth, physiology, yield, and quality attributes and comprehensive evaluation of wolfberry (*Lycium barbarum* L.). Front. Plant Sci..

[39] Chen T., Liu L., Zhou Y.L., Zheng Q., Luo S.Y., Xiang T.T., Zhou L.J., Feng S.L., Yang H.Y., Ding C.B. (2023). Characterization and comprehensive evaluation of phenotypic characters in wild *Camellia oleifera* germplasm for conservation and breeding. Front. Plant Sci..

[40] Desta K.T., Choi Y.M., Shin M.J., Yoon H., Wang X.H., Lee Y., Yi J., Jeon Y.A., Lee S. (2023). Comprehensive evaluation of nutritional components, bioactive metabolites, and antioxidant activities in diverse sorghum (*Sorghum bicolor* (L.) Moench) landraces. Food Res. Int..

[41] Yang H.Y., Duan Y.K., Wei Z.W., Wu Y.Q., Zhang C.H., Wu W.L., Lyu L.F., Li W.L. (2022). Integrated physiological and metabolomic analyses reveal the differences in the fruit quality of the blueberry cultivated in three soilless substrates. Foods.

[42] Liu X.Y., Wu R.M., Bulley S.M., Zhong C.H., Li D.W. (2022). Kiwifruit MYBS1-like and GBF3 transcription factors influence l-ascorbic acid biosynthesis by activating transcription of GDP-L-galactose phosphorylase 3. New Phytol..

[43] Lin C., Zhang X.Q., Sun P.L., Liu S., Mao J.L., Lei H.X., Ma Y.H., Xu W.Z., Pan F.J., Chen M.P. (2025). Genetic Diversity and Population Structure of *Actinidia eriantha* by ITS Sequences. Nat. Prod. J..

[44] Guo R., Landis J.B., Moore M.J., Meng A., Jian S., Yao X., Wang H. (2017). Development and application of transcriptome-derived microsatellites in *Actinidia eriantha* (Actinidiaceae). Front. Plant Sci..

[45] Yang T., Huang Y.J., Li S.H., Ren D., Cui J.X., Pang B., Yu S., Gao W.W. (2021). Genetic diversity and comprehensive evaluation of phenotypic traits in sea-Island cotton germplasm resources. Sci. Agric. Sin..

